# Sublethal Effects in Pest Management: A Surrogate Species Perspective on Fruit Fly Control

**DOI:** 10.3390/insects8030078

**Published:** 2017-07-29

**Authors:** John E. Banks, Roger I. Vargas, Azmy S. Ackleh, John D. Stark

**Affiliations:** 1 Undergraduate Research Opportunities Center, California State University, Monterey Bay, Seaside, CA 93955, USA; 2 Daniel K. Inouye, U.S. Pacific Basin Agricultural Research Center, Agricultural Research Service, United States Department of Agriculture, 64 Nowelo St., Hilo, HI 96720, USA; roger.vargas@ars.usda.gov; 3 R.P. Authement College of Sciences , University of Louisiana at Lafayette, 201 Oliver Hall, P.O. Box 43649, Lafeyette, LA 70504, USA; ackleh@louisiana.edu; 4 Washington State University Puyallup Research & Extension Center, 2606 W. Pioneer Ave, Puyallup, WA 98371, USA; starkj@wsu.edu

**Keywords:** Lefkovitch matrix, surrogate species, Tephritidae

## Abstract

Tephritid fruit flies are economically important orchard pests globally. While much effort has focused on controlling individual species with a combination of pesticides and biological control, less attention has been paid to managing assemblages of species. Although several tephritid species may co-occur in orchards/cultivated areas, especially in mixed-cropping schemes, their responses to pesticides may be highly variable. Furthermore, predictive efforts about toxicant effects are generally based on acute toxicity, with little or no regard to long-term population effects. Using a simple matrix model parameterized with life history data, we quantified the responses of several tephritid species to the sublethal effects of a toxicant acting on fecundity. Using a critical threshold to determine levels of fecundity reduction below which species are driven to local extinction, we determined that threshold levels vary widely for the three tephritid species. In particular, *Bactrocera dorsalis* was the most robust of the three species, followed by *Ceratitis capitata*, and then *B. cucurbitae*, suggesting individual species responses should be taken into account when planning for area-wide pest control. The rank-order of susceptibility contrasts with results from several field/lab studies testing the same species, suggesting that considering a combination of life history traits and individual species susceptibility is necessary for understanding population responses of species assemblages to toxicant exposure.

## 1. Introduction

Understanding how exposure to chemical compounds affects the population dynamics of economically important pest species is critical in developing effective integrated pest management schemes. More than two decades ago, the Food Quality Protection Act [1] resulted in the review of nearly 10,000 pesticide uses in American agriculture. The ensuing loss of the use of many pesticides in turn has spurred on the development of a new suite of chemical compounds, many of which were introduced as safer, more selective forms of pest control. Yet the methodologies used in pesticide risk assessment have lagged behind, with many such assessments relying heavily on static measures such as the LD_50_/LC_50_. These measures are useful in ranking the toxicity of a wide range of toxicants, and, together with measures of exposure, can provide a framework for understanding potential effects on target and non-target organisms. However, by their very definition static metrics cannot capture longer-term population outcomes—specifically those stemming from susceptibility and vital rates that differ across life stages/ages, or sublethal effects of toxicants such as reductions in fecundity [2,3,4]. Population models—especially those incorporating parameter values from field or lab experiments—have yielded much needed insight into more nuanced effects of toxicants such as pesticides [5,6,7,8,9].

A common method of anticipating the effects of a disturbance such as a toxicant on target and non-target organisms is to test the effects on one species and extrapolate to a wider range of closely-related species. This so-called “surrogate species” approach is used widely in conservation science, and also has applications in biological control, especially for the protection of natural enemies [2]. This approach has high potential for misleading results, namely because differences in life-history strategies/vital rates may lead to very different population trajectories in the long run [7].

We use a simple mathematical model here to explore the utility of such a surrogate species approach in assessing the efficacy of pesticides in controlling three economically important fruit fly species. We parameterized a matrix model using life table data derived in the laboratory for three Tephritid species found commonly in orchards: Mediterranean fruit fly, *Ceratitis capitata* (Wiedemann), Oriental fruit fly, *Bactrocera dorsalis* (Hendel), and Melon fly, *B. cucurbitae* (Coquillett). Spinosad-based sprays are commonly used to control all three of these species [10]—a spinosad-based hydrolysed protein bait (referred to as GF-120), for instance, has been used to control *B. dorsalis* [11] and *B. cucurbitae* [12] in area-wide programs in Hawaii. In some area-wide eradication schemes, multiple species may be exposed to the same toxicants, with the expectation that populations of different species may respond similarly; in such cases, a surrogate species perspective may be a useful approach to optimizing pest management. We explore here just such an approach, incorporating lab-derived life history data into a simple mathematical model in order to compare species’ responses to pesticide exposure.

## 2. Materials and Methods

### 2.1. Model

We used a standard Lefkovitch matrix model [13] to project population growth for three different tephritid species, each with four life stages (e.g., egg, larva, pupa, and adult). In the most general form of this model, the number of individuals in each of these four stage classes is denoted by $x_{i}$ for *i =* (1, 2, 3, 4) with the population expressed as a vector $X={\left[\begin{array}{cccc} x_{1}, & x_{2}, & x_{3}, & x_{4} \end{array}\right]}^{T}$. Then the population growth may be described by the matrix equation:

(1) $$X\left(t+1\right)=\left[\begin{array}{c} x_{1}\left(t+1\right)\\ x_{2}\left(t+1\right)\\ x_{3}\left(t+1\right)\\ x_{4}\left(t+1\right) \end{array}\right]=\left[\begin{array}{cccc} 0 & 0 & 0 & f_{4}\\ a_{1} & 0 & 0 & 0\\ 0 & a_{2} & 0 & 0\\ 0 & 0 & a_{3} & a_{4} \end{array}\right]\left[\begin{array}{c} x_{1}\left(t\right)\\ x_{2}\left(t\right)\\ x_{3}\left(t\right)\\ x_{4}\left(t\right) \end{array}\right]=AX\left(t\right)$$

where the $a_{i}$ represent the rate of individuals surviving to the next stage ($0<a_{i}<1,\;i=1,\;2,\;3$ and $0\leq a_{4}<1$), and $f_{4}$ denotes the reproductive rate of the 4th (adult) life stage. If the dominant eigenvalue (λ) of the transition matrix (A) is greater than one, then the population will grow [14,15]. Elsewhere [16], we have derived an expression relating the dominant eigenvalue to the net reproductive rate of the population, $R_{0}$—that is, the number of offspring produced by one individual during the course of its lifetime. This rate is given by:

(2) $$R_{0}=\frac{f_{4}a_{1}a_{2}a_{3}}{1-a_{4}}=f_{4}a_{1}a_{2}a_{3}\left(1+a_{4}+a_{4}^{2}+a_{4}^{3}+\cdots \right)$$

For this system, $R_{0\;}$> 1 corresponds to the dominant eigenvalue (λ) of the matrix A being greater than one, which represents a growing population. Conversely, $R_{0}$ < 1 corresponds to λ < 1, representing the population going to extinction [17]. Biologically speaking, this means that as long as each individual reproduces at a level slightly higher than replacement ($R_{0}\;$> 1), the population will grow.

### 2.2. Model Parameterization: Tephritid Life History Data

We simulated population outcomes using life table data for each of the three tephritids:

Mediterranean fruit fly *Ceratitis capitata* (Wiedemann), Oriental fruit fly *Bactrocera dorsalis* (Hendel), and Melon fly *B. cucurbitae* (Coquillett). *C. capitata*, *B. dorsalis* and *B. cucurbitatae* are global pest of soft fruits and vegetables with 361, 627 and 136 confirmed hosts, respectively, under field conditions [18].

Daily vital rates for all three tephritids, which are published elsewhere [10] were transformed into stage rates (egg, larval, pupal and adult) for this study. Daily survival rates were converted to stage survival rates by dividing the final number of survivors at the end of the stage by the initial number of individuals alive at the beginning of the stage (Table 1). Stage length was different for some of the stages and varied across species (1 day for egg, 6–7 days for larval, 9 days for pupal, 106–290 days for adult). The total number of offspring were summed over the number of days in the adult stage to generate fecundity rates. 

### 2.3. Calculation of Critical Extinction Thresholds

Using life history data specific to each of the three tephritid species, we used Equation (2) to simulate the sublethal effects of pesticide exposure on population persistence. We did this by systematically reducing the fecundity of each species in the model and calculating the ensuing population projections. In this way, we were able to calculate the levels beyond which reductions in fecundity due to pesticide exposure would render the different tephritid species extinct. For this calculation, we denoted the net reproductive number of each species by $R_{0}$. We began by assuming that in the absence of a toxicant the tephritid species survives and thus has a net reproductive rate greater than 1. Furthermore, we assumed that there is a critical level of toxicant (pesticide) exposure that corresponds to the case in which the net reproductive rate for the tephritid species drops below one and hence goes to extinction. We let $R_{0}=1+\varepsilon >1$ where ε is a positive number, and then denoted by $R_{0}^{T}$ the net reproductive rate when the tephritid species is exposed to pesticides, reflecting the way in which the toxicant influences the net reproductive rates. We next related this measure to the life history traits (the measurable entities in our transition matrix A). For simplicity, we assumed that toxicants mainly influence reproduction rates; with further analysis we could extend this method to the case where survivorship rates were also influenced. We also assumed that the toxicant reduced the fecundity rates of each of the three tephritid species in a similar fashion. We denoted the percent reduction in fecundity rate by δ (a positive number between 0 and 1; 0 is zero percent and 1 is 100%). Thus, the fecundity rate for a tephritid species with the presence of toxicants could be expressed by $f_{4}^{T}=f_{4}\left(1-\delta \right)$. Using the expression for the net reproductive rate derived above in Equation (2), the net reproductive rate for each species with toxicant present became:

(3) $$R_{0}=\frac{a_{1}a_{2}a_{3}f_{4}^{T}}{1-a_{4}}=\frac{a_{1}a_{2}a_{3}f_{4}\left(1-\delta \right)}{1-a_{4}}$$

Substituting in $R_{0}=1+\varepsilon$ in Equation (3), we derived the following critical criteria for persistence of each tephritid species:

(4) $$\frac{a_{1}a_{2}a_{3}f_{4}\delta }{1-a_{4}}<\varepsilon$$

where ε is the difference between the net reproductive rate of the tephritid species and 1 (i.e., it describes how far the net reproductive rate is from 1 when no toxicants are present). (Please confirm)

### 2.4. Pest Management: Tephritid Species’ Responses to Pesticides

The physiological effects of pesticides and other toxicants are routinely extrapolated from one species to another species, especially for economically important arthropods. We apply this perspective to management of the suite of tephritid species in order to predict how reliable the response of one species to pesticide exposure may be extrapolated to the other species. To do this, we applied the criteria derived above in a comparison of extinction thresholds for the three tephritid species. In particular, we illustrated how one may use equations (Equations (3) and (4) above) to compare the robustness of population projections among these three species. For each species, we plugged in the life history parameter values (corresponding to transition matrix elements) directly into Equation (4) (Table 1).

Because $R_{0}=1+\varepsilon$, we can solve for the right hand side of Equation (4) by evaluating $\varepsilon =R_{0}-1$. For each of the three tephritid species, we evaluated Equation (4) for a pesticide that reduces fecundity in increments of 10%. We ran the simulation for a 10% reduction and noted the population outcome, then ran it again for a 20% reduction, and so on, each time noting the population response until it was driven to local extinction. For each species, we calculated a threshold level of fecundity reduction, above which the population went extinct. This enabled us to assess the relative robustness of each of the three species with respect to population outcomes after pesticide exposure.

## 3. Results

**Figure 1:**
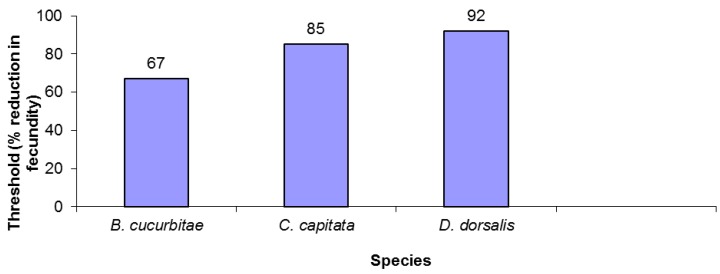
Extinction thresholds for three tephritid species.

The three tephritid species varied in fecundity reduction thresholds. *B. cucurbitae* was the least robust, driven to extinction after only a 67% reduction in fecundity (Figure 1). *B. dorsalis* was over 35% more robust, withstanding over a 90% decrease in fecundity before going extinct (Figure 1). Finally, *C. capitata* had an intermediate level of susceptibility, persisting with fecundity reductions of up to 85%.

## 4. Discussion and Conclusions

Population outcomes for the three tephritid species, generated from matrix model projections using life table data, revealed that different species responded quite differently to pesticide disturbance. This illustrates that in general we cannot anticipate the effects that sublethal toxicological insults may have on one species based on the results of another—even when those species are members of the same family. This of course has extensive implications for the application of pesticides in polycultures that may harbor a suite of pest species, and is particularly pertinent to the inclusion of pesticide sprays in IPM for area-wide control of orchard pests [19,20]. A surrogate species approach to pest management requires more attention to detail—including comparisons of specific vital rates derived from life table experiments among similar species.

The results of our study strongly contrast with those of a suite of toxicological studies conducted previously on these same three tephritid species. LC_50_ values for the same species exposed to Spinosad were nearly the inverse of those we found, with *B. cucurbitae* the least susceptible [21]. On the other hand, LC_50_ data generated for Avermectin B1, indicate that *C. capitata* is the most robust species by far, followed distantly by *B. cucurbitae* and *B. dorsalis* [22]. Furthermore, *C. capitata* was the least robust species in LC_50_ tests of Cyromazine and Diazinon [23]. Short-term measures of toxicity, such as the LD/LC_50_ may completely miss the susceptibility of a species at the population level over longer time frames, especially those spanning several generations. Some species may be more susceptible to some toxicants than others, which may in those cases mitigate their inherent advantage in, say, responding to sublethal effects (e.g., *B. dorsalis*). That is, while a particular species may be very robust, they may also be very susceptible to a specific chemical, thus offsetting any advantages attributable to life history/vital rates. Taken together, our results suggest that differences in vital rates as well as differential susceptibility to pesticides need to be considered when evaluating pest control in orchard IPM programs.

## Figures and Tables

**Table 1:** Life history values for three tephritid species used in model.

Species	*C. capitata*		*B. cucurbitae*		*B. dorsalis*	
Stage	$a_{i}$	$f_{i}$	$a_{i}$	$f_{i}$	$a_{i}$	$f_{i}$
Egg	0.96	0	0.86	0	0.92	0
Larva	0.916667	0	0.918605	0	0.771739	0
Pupa	0.943182	0	0.911392	0	0.859155	0
Adult	0.012048	8.434882	0.013889	4.232877	0.016393	22.13273
